# Ultraviolet Response in Coplanar Silicon Avalanche Photodiodes with CMOS Compatibility

**DOI:** 10.3390/s22103873

**Published:** 2022-05-20

**Authors:** Qiaoli Liu, Li Xu, Yuxin Jin, Shifeng Zhang, Yitong Wang, Anqi Hu, Xia Guo

**Affiliations:** State Key Laboratory for Information Photonics and Optical Communications, School of Electronic Engineering, Beijing University of Posts and Telecommunications, Beijing 100876, China; liuqiaoli@bupt.edu.cn (Q.L.); xulinora@bupt.edu.cn (L.X.); jinyuxin@bupt.edu.cn (Y.J.); zsfeng@bupt.edu.cn (S.Z.); wytong@bupt.edu.cn (Y.W.); anqihu@bupt.edu.cn (A.H.)

**Keywords:** avalanche photodiodes (APDs), UV response, responsivity, gain, avalanche buildup

## Abstract

Highly sensitive ultraviolet (UV) photodetectors are highly desired for industrial and scientific applications. However, the responsivity of silicon photodiodes in the UV wavelength band is relatively low due to high-density Si/SiO_2_ interface states. In this paper, a coplanar avalanche photodiode (APD) was developed with a virtual guard ring design. When working in Geiger mode, it exhibited a strong UV response. The responsivity of 4 × 10^3^ A/W (corresponding to a gain of 8 × 10^6^) at 261 nm is measured under the incident power of 0.6 μW with an excess bias of 1.5 V. To the best of our knowledge, the maximum 3-dB bandwidth of 1.4 GHz is the first report ever for a Si APD when working in the Geiger mode in spite of the absence of an integrated CMOS read-out circuit.

## 1. Introduction

The highly sensitive ultraviolet (UV) photodetector is intensively demanded owing to its great potential in applications of scientific analysis, industrial systems, fire warning, etc. [1,2,3,4,5]. Recently, high-sensitive UV photodetectors are of great interest in the area of quantum communications and quantum ghost imaging [6,7]. Si photodetector is the workhorse in the wavelength range from 300 to 1100 nm with the distinctive advantages of high responsivity (*R*), low noise, and low cost. Especially, its large-scale array can be fabricated by CMOS technology with easy integration of read-out circuits, which is an important feature for imaging applications. However, its photoresponse is relatively low in the UV wavelength band because high Si/SiO_2_ interface state density results in strong interface non-radiation recombination [8].

In order to improve the UV response of Si photodiode, pure boron (PureB) technology is developed for an ultrathin and steep junction [9,10], in which the photogenerated carriers are drifted quickly to the depletion layer under the action of steep junction in order to form the detectable photocurrent. The PureB layer provides an effective p^+^-doping of the semiconductor surface to form a nanometer-thin p^+^-n junction and a light-entrance window as well. The external quantum efficiency of the photodiode can reach as high as 96% (0.2 A/W) for the incident light of 256 nm [10].

To pursue the ultimate sensitivity, UV avalanche photodiode (APD) is highly desired. Si APD with PureB technology demonstrated relatively high responsivity only at the wavelength ranging from 330 to 370 nm when operated in the Geiger mode without applying a capping filter at room temperature [11]. Recently, backside-illuminated (BSI) APDs with 650 nm-thin Si body based on SOI technology were developed. Peak photon detection efficiency (PDE) of 22.13% was achieved at a wavelength of 423 nm for the excess bias ($V_{ex}$) of 3 V with a dark count rate (DCR) of 156.8 Hz/μm^2^ at room temperature. A significant expansion of the UV sensitivity down to a wavelength of 291 nm was achieved with a responsivity of 0.05 A/W at 4 V [12]. Though it was believed that the detection efficiency of this ultrathin BSI APD in UV wavelength regime down to 291 nm was the best result ever reported for Si-based BSI APD, it is still a great challenge to realize a UV Si APD with high sensitivity.

In this paper, a coplanar Si APD structure was developed with a virtual guard ring. When working in Geiger mode, it exhibited a strong UV response. The Si APD is characterized by low dark current and low breakdown voltage at room temperature. The responsivity of 4 × 10^3^ A/W (corresponding to a gain of 8 × 10^6^) at 261 nm is measured under the incident power of 0.6 μW with an excess bias of 1.5 V. To the best of our knowledge, the frequency response of 1.4 GHz is the first report ever for Si APDs when working in the Geiger mode in spite of the absence of an integrated CMOS read-out circuit.

## 2. Design and Results

**Figure 1:**
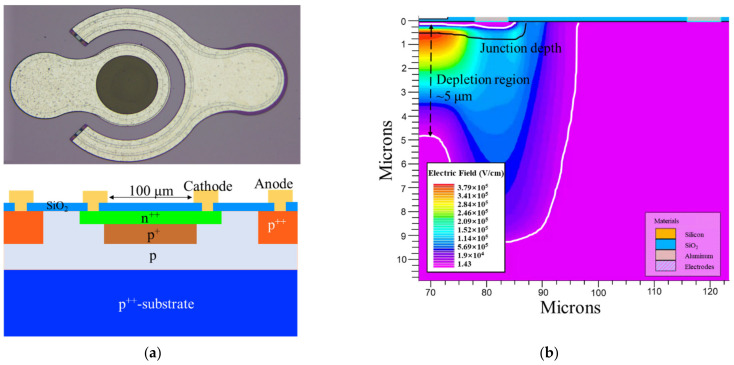

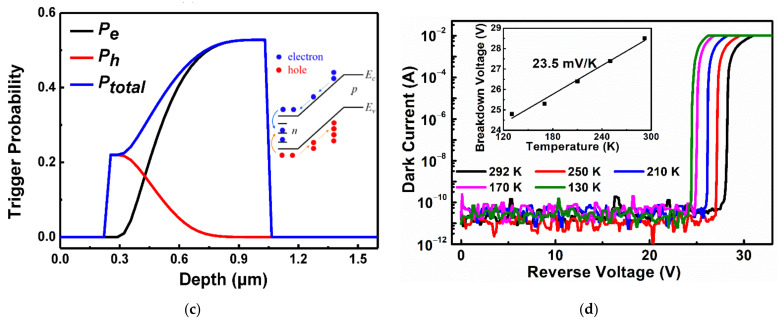
Design and electrical characteristics of the APD. (**a**) Upper panel: an optical microscope of the 100-μm Si APD. Bottom panel: the cross-sectional view of the device. (**b**) Simulated electric field at breakdown with a depletion region width of ~5 μm. (**c**) Probability to breakdown is simulated at the *V_ex_* of 2.5 V by Sentaurus TCAD. Inset: Illustration of photogenerated carrier transit process under the external bias. Part of photogenerated holes trigger the avalanche, while most of photogenerated electrons are trapped at the Si/SiO_2_ interface. (**d**) Measured reverse dark current-voltage (I-V) curves at temperature from 130 K to 292 K in steps of about 40 K, respectively, for a Si APD with a diameter of 100 μm. Inset: Temperature dependence of *V_br_*, which is calculated to be 23.5 mV/K for the temperature ranging from 130 K to 292 K.

The upper panel of Figure 1 shows an optical microscope of the Si APD with a diameter of 100 μm. It is basically an n-on-p structure realized on a p-type epilayer, as depicted in the bottom panel of Figure 1. The p^+^ multiplication layer is formed by the implantation method, followed by an annealing process. Then the p^++^ and n^++^ ohmic contact layers are formed by ion implantation. The virtual guard ring technique is utilized in this work, in which the high field is mainly concentrated in the center, and the edge breakdown is suppressed in such a coplanar structure. The distance between the anode and the edge of the n^++^ layer is 30 μm with an anode width of 20 μm. Regrown CMOS-grade dry oxygen oxidation SiO_2_ was utilized as an antireflection layer to improve the Si/SiO_2_ interface quality.

Figure 1b shows a cross-sectional electric-field distribution on the right half of the designed Si APD simulated by Silvaco TCAD. The doping profile provides a high built-in electric field intensity (*E*) of ~4 × 10^5^ V/cm at breakdown voltage with a depletion region width of ~5 μm. The photogenerated holes initiate the impact ionization under the action of the high electric field and avalanche multiplication in Geiger mode.

The avalanche trigger probability is simulated at a *V_ex_* of 2.5 V by Sentaurus TCAD, as shown in Figure 1c. $P_{e}$ and $P_{h}$ are the avalanche trigger probabilities initiated by electrons and holes across the depletion region, respectively. $P_{total}$ is the total electron-hole pairs’ avalanche trigger probability. Though the avalanche probability of electrons is higher, limited by the penetration length for 261 nm light in Si (~5 nm), the hole‘s trigger probability plays a major role in this structure, with the maximum occurring at the edge of the n-side space-charge region. As illustrated in the inset, photogenerated electron-hole pairs within the dead region will recombine. Only part of the holes can have the possibility to diffuse to the edge of the depletion region and then trigger the impact ionization within their lifetime.

Figure 1d shows the temperature dependence of reverse dark current-voltage (I-V) characteristics, which were measured on the temperature-controlled probe station from room temperature down to 130 K in steps of about 40 K. The proposed Si APD has low dark current ($I_{d}$), which is lower than 0.1 nA. $V_{br}$ is 28.5 V at room temperature, which is determined when $I_{d}$ reaches 100 μA in this work. To be noted, the large noise before breakdown is from the background and the vibration of the refrigeration system for the probe station, on which the chip under test was put directly. The inset of Figure 1d presents that $V_{br}$ increases with temperature because lattice scattering becomes more intensive with temperature hence the phonon scattering rate. The temperature coefficient of $V_{br}$ is calculated to be 23.5 mV/K for the temperature ranging from 130 K to 292 K. Carriers will experience lattice scattering and impact ionization scattering during the drift process under the action of the electric field. Only when the energy is above the threshold energy, is it possible for the carriers to trigger the impact ionization process, and then the avalanche process is buildup when it is large enough. The temperature coefficient of $V_{br}$ has a relationship with the phonon scattering degree. A smaller temperature coefficient of $V_{br}$ means the breakdown voltage is less sensitive to a higher temperature, and so are the ionization coefficients. Thus the carriers will scatter fewer phonons before ionization and acquire the ionization threshold energy much faster, which shortens the avalanche buildup process. So the relatively small temperature coefficient of $V_{br}$ in our experiment, compared with the reported data [13,14,15], indicates the carriers acquire the ionization threshold energy much faster, and scatter fewer phonons before ionization, then a faster avalanche buildup process [16].

**Figure 2:**
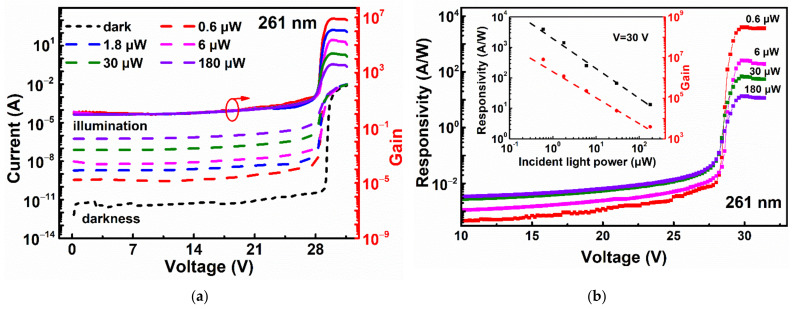

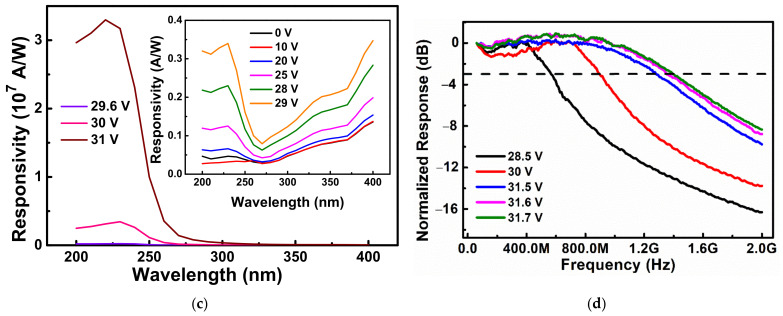
Photoresponse and frequency response of the APD. (**a**) Measured reverse current-voltage-gain curves of the Si APD at different incident light power (0.6 μW, 1.8 μW, 6 μW, 30 μW, 180 μW) of 261 nm. Dark current, photocurrent, and gain are represented by black short dot, colored dash line and corresponding colored solid line, respectively. (**b**) The responsivity as a function of reverse bias under the illumination of 261 nm. Inset: Extracted responsivity and gain at 30 V from Figure 2a and the corresponding fitting results. (**c**) Spectral responsivity measurement results biased above breakdown for the incident light wavelength from 200 nm to 400 nm. Inset: Spectral responsivity biased before breakdown. (**d**) Measured optical modulation frequency response at different reverse biases when the incident light wavelength is 850 nm for the 100-μm Si APD.

Figure 2a shows the reverse I-V characteristics with and without light illumination at room temperature. After calibration, the incident light power (*P*) of 0.6 μW, 1.8 μW, 6 μW, 30 μW, and 180 μW are achieved, respectively, with the laser wavelength of 261 nm, which is attenuated by a series of ultraviolet neutral density filters. The photocurrent, defined by subtracting dark current from the current under illumination ($I_{p}$), increases with the incident light power before breakdown due to the increase of photogenerated carrier. After breakdown, the photocurrent for all the incident light power increases suddenly. Avalanche gain (*M*) is defined as the ratio of photocurrent to that at a reference bias $V_{0}$ for *M* = 1, which can be expressed as [17]

(1) $$M=\frac{I_{p}\left(V\right)-I_{d}\left(V\right)}{I_{p}\left(V_{0}\right)-I_{d}\left(V_{0}\right)},$$

$V_{0}$ is set to be 10 V in this work. The gain curves increase sharply with bias due to the avalanche multiplication process even when the bias is larger than $V_{br}$.

Figure 2b presents the responsivity *R* as a function of external bias under the illumination of 261 nm. At 10 V, the responsivity is around 0.5 mA/W at an incident power of 0.6 μW with a quantum efficiency of 0.24%. After breakdown, the responsivity increases also sharply. Inset shows the responsivity and gains results at 30 V under different incident light power. The maximum gain of 8 × 10^6^ is obtained at the incident light power of 0.6 μW with *R* of 4 × 10^3^ A/W, which can be expressed as

(2) $$R=\frac{I_{p}-I_{d}}{P},$$

The fitting result shows that

(3) $$R=1936\cdot P^{-0.98},$$

(4) $$M=2\times 10^{6}\cdot P^{-1.28},$$

respectively, both *R* and *M* are approximately inversely proportional to the incident light power *P* after breakdown due to the reduction of electric field intensity in the depletion region. The effective electric field within the depletion region decreases with the incident light power, which decreases the avalanche probability. The deviation of *R* and *M* from inversion relation is possibly caused by measurement error. A brief UV response comparison with other reported reports is shown in Table 1.

Figure 2c shows the spectral responsivity measurement results from 200 nm to 400 nm under the bias above breakdown by Acton SpectraPro 2500i monochromator (Acton Research Co., Acton, MA, USA). The power of the spectral light source is obtained by a commercial Si photodiode according to the definition of *R.* The light source power increases with the wavelength from 0.1 nW to 46 nW. It can be seen the responsivity enhances greatly for the wavelength lower than 300 nm. The peak responsivity of 3.3 × 10^7^ A/W is obtained at around 220 nm when biased at 31 V. Compared with the data in Figure 2b, ~4 orders of magnitude enhancement in responsivity are caused by ~4 orders of magnitude reduction of the incident light power. Inset shows the spectral responsivity measurement results biased before breakdown. All the responsivity curves increase with bias with a peak wavelength of around 220 nm. Besides, the DCR of the device is also measured with a passive quenching circuit. The DCR is high and even increases to 100 kHz. It may be attributed to the relatively low quality of the used substrate material, which causes high trap-assisted tunneling at large electric field strengths [22].

Figure 2d shows the optical modulation frequency response $S_{21}$ of Si APD at different biases measured by using Vector Network Analyzer (VNA) with a 25 Gbit/s 850 nm vertical-cavity surface-emitting laser (VCSEL, New Focus, San Jose, CA, USA) as the light source. Prior to the measurement, the on-wafer short-open-load calibration without illumination was carried out for measurement accuracy. After breakdown, the 3-dB bandwidth increases abruptly due to an rf enhancement effect. This rf peaking is attributed to inductive component generation in the avalanche region of an APD [23,24]. The maximum 3-dB bandwidth obtained is 1.4 GHz when biased at 31.7 V. The gain under the illumination of 850 nm is about 4 × 10^6^ with the same incident power of optical frequency modulation measurement. The corresponding gain-bandwidth product can be as high as ~6 × 10^6^ GHz.

Generally, the frequency response of a device is determined by the transit time $\tau_{transit}$, diffuse time $\tau_{diffuse}$, and *RC* time $\tau_{RC}$. However, for an avalanche device, the avalanche buildup time $\tau_{buildup}$ can not be omitted, which is the time interval from the injection of a carrier into the depletion region to the formation of a measurable avalanche multiplication current [25]. $\tau_{buildup}$ is strongly dependent on the electric field profile of the reverse-biased depletion region [26]. Hence, the response time *τ* can be derived to be

(5) $$\tau =\sqrt{\tau_{buildup}^{2}+\tau_{transist}^{2}+\tau_{RC}^{2}+\tau_{diffuse}^{2}},$$

where the p-type layer is completely depleted without the carrier diffusion process, $\tau_{diffuse}$ can be omitted here.

**Figure 3:**
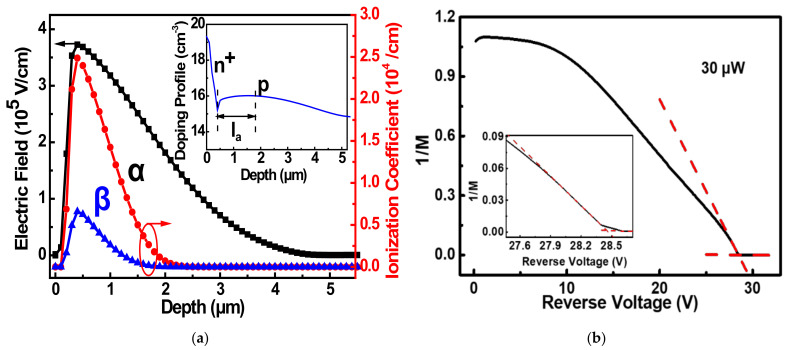
Electric field simulation and 1/*M* versus reverse voltage. (**a**) *α* (red symbol), *β* (blue symbol), and electric field (black symbol) distribution at *V_br_*. Inset: Extracted doping profile of the structure. (**b**) 1/*M* as a function of reverse bias at 30 μW (black line) and fitting curves (red dash line) from Figure 2a. Inset: Enlarged view at the breakdown.

Avalanche buildup time can be expressed by

(6) $$\tau_{buildup}=\tau_{1}\cdot M,$$

where $\tau_{1}$ is the intrinsic response time [27,28], and is considered to be closely related to the mean free time between ionization collisions.

(7) $$\tau_{1}=N\cdot k_{eff}\cdot l_{a}/v_{s},$$

where is obtained by Takao Kaneda [29,30], and $k_{eff}$ can be expressed by

(8) $$k_{eff}=\beta /\alpha {|}_{max},$$

where $l_{a}$ is the avalanche region width, $v_{s}$ is the carrier saturation velocity, and *β* and *α* are the hole and electron collision ionization coefficients, respectively. *N* is a number varying slowly from 1/3 to 2 as *β*/*α* varies from 1 to 10^−3^, respectively [29,30]. All these parameters are from the simulation results of the electric field distribution at $V_{br}$ with the corresponding ionization coefficient by Silvaco TCAD software, as shown in Figure 3a. Both *α* and *β* show an exponential relationship with the electric field, which are

(9) $$\alpha =6.97\times 10^{5}\cdot e^{-1.24\times 10^{6}/E},$$

(10) $$\beta =1.47\times 10^{6}\cdot e^{-2.01\times 10^{6}/E},$$

respectively. The maximum electric field $E_{M}$ is 3.7 × 10^5^ V/cm according to the simulation and then $k_{eff}$ is 0.265. From the doping profile of this simulation structure shown in the inset of Figure 3a, it can be seen that $l_{a}$ is about 1.4 μm and *N* is 1.1 from Takao Kaneda’s calculation [30]. Then $\tau_{1}$ of 4.08 ps can be obtained.

Figure 3b presents the data from Figure 2a of 1/*M* versus the reverse bias with the incident light power of 30 μW. The intercept of the voltage axis should be the theoretical $V_{br}$ (28.5 V) under illumination, and then the corresponding gain is 156, according to Shockley’s gain empirical formula [31]

(11) $$1/M=n\left(1-V/V_{br}\right).$$

where *n* is a constant which depends on the material. Hence, $\tau_{buildup}$ is 636.48 ps. $\tau_{transit}$ is estimated to be 50 ps with saturated velocity because the p-epi layer is completely depleted without the carrier diffusion process. $\tau_{RC}$ is estimated to be 498 ps according to our capacitance-voltage and I-V measurement results for this Si APD. Hence, the response time *τ* is around 809.70 ps, which can match the optical modulation frequency response results.

## 3. Discussion

In order to figure out the UV response mechanism of our device, APD structures with the temperature coefficient of $V_{br}$ of 0.5~0.7 V/K from different sponsors are selected for experiment comparison. However, no UV response signal was obtained. Also, for the APDs with the same multiplication junction, no matter lateral or vertical structure, it was easy to obtain a clear and obvious UV response current only after breakdown. It is known that such a multiplication structure with a low temperature coefficient of $V_{br}$ is typically for high-precision time-of-flight measurement due to its high timing resolution. We attributed the UV response to such a special multiplication structure with a small temperature coefficient of $V_{br}$.

The temperature coefficient of $V_{br}$ is an indicator of phonon scattering degree or energy acquisition efficiency from the high electric field. Within the same multiplication length, if carriers can acquire the impact ionization threshold energy faster and suffer from fewer phonon scattering, that is short avalanche buildup time, the temperature dependence of the ionization coefficient will be consequently reduced, which results in a smaller temperature coefficient of $V_{br}$. High timing performance is resulted from the inhibited avalanche randomness process.

As analyzed above, avalanche buildup time can not be omitted due to its complicated impact ionization and accumulation process. The fast avalanche buildup process indicates photogenerated holes have a chance to trigger the impact ionization within their lifetime after diffusing to the edge of the depletion region. While the lifetime of holes is determined by the electron trapped by the interface states, which shortens the effective lifetime of these photogenerated carriers greatly [32]. If a photogenerated hole triggers the avalanche process within the lifetime of the photogenerated electron, the UV signal can be enlarged and detected. That’s the reason that UV response signal can be obtained by only Si APDs with a low temperature coefficient of $V_{br}$.

## 4. Conclusions

A UV Si APD with a photosensitive diameter of 100 μm is demonstrated based on lateral structure. The static and dynamic characterizations were carried out for understanding the UV response in Si APDs. The temperature coefficient of $V_{br}$ is 23.5 mV/K and a low $V_{br}$ of 28.5 V at room temperature. The maximum responsivity of 4 × 10^3^ A/W (corresponding to a gain of ~8 × 10^6^) is achieved at a wavelength of 261 nm under the incident power of 0.6 μW after breakdown. The maximum 3-dB bandwidth obtained is 1.4 GHz when biased at 31.7 V under the 850 nm laser illumination. Then the avalanche buildup time of the Si APD used in this work has been estimated to be ~640 ps. The UV response of the Si APD is attributed to the fast avalanche buildup process. This work helps understand how to obtain a high UV response in Si APDs.

## Figures and Tables

**Table 1:** The UV response comparison with other reported papers.

Reference	Structure	Responsivity	Wavelength	Bias
[10]	photodiode	0.2 A/W	256 nm	2 V
[18]	photodiode	0.06 A/W	260 nm	0 V
[19]	photodiode	0.1 A/W	255 nm	0 V
[20]	photodiode	0.2 A/W	200 nm	0 V
[21]	APD	0.18 A/W	275 nm	4 V
[12]	APD	0.05 A/W	291 nm	4 V
		~10^3^ A/W		V_ex_ = 0.5 V
This work	APD	0.5 mA/W	261 nm	10 V
		4 × 10^3^ A/W		V_ex_ = 1.5 V

## Data Availability

Not applicable.
